# Supplementary material for: Collaborative intelligence in AI: Evaluating the performance of a council of AIs on the USMLE
Source: PLOS Digit Health. 2025 Oct 9;4(10):e0000787. doi: 10.1371/journal.pdig.0000787 (PMC12510544; doi:10.1371/journal.pdig.0000787)
Supplement: S2 File — (ZIP) [file pdig.0000787.s002.zip › S2 Data Raw data files containing unprocessed question inputs and LLM outputs including transcripts of deliberations/STEP3_Question-42-1_Type-MC-NJ.docx]

**Facilitator** **[Round 1]:** A 31-year-old woman, gravida 3, para 2, who is at 32 weeks' gestation, is admitted to the hospital because of a 1-week history of progressive dyspnea and wheezing. She says her heart is ""racing"" and she is coughing up a small amount of blood-streaked sputum. Medical history is significant for hypothyroidism, for which she takes levothyroxine. An intravenous catheter is placed. Vital signs are temperature 36.9°C (98.4°F), pulse 132/min, respirations 32/min, and blood pressure 135/78 mm Hg. Pulse oximetry on 100% oxygen via nasal cannula shows an oxygen saturation of 92%. Auscultation of the lungs discloses decreased breath sounds at the bases with expiratory crackles bilaterally. Cardiac examination discloses an irregularly irregular rhythm, an indistinct point of maximal impulse, and a loud S1. A grade 3/6, low-pitched, diastolic, rumbling murmur is audible at the apex; a distinct snapping sound precedes the murmur. Fetal heart rate is 144/min. Which of the following is the most likely cause of the findings in this patient? (A) Atrial septal defect with development of pulmonary hypertension (B) Chronic mitral regurgitation secondary to rheumatic heart disease (C) Coarctation of the aorta (D) Congenital aortic stenosis (E) Mitral stenosis complicated by atrial fibrillation

**AI_D:** RESTATING THE QUESTION: A pregnant woman is presenting symptoms such as dyspnea, wheezing, and coughing up blood-streaked sputum which indicate potential cardiac complications. The examination findings point towards possible heart defects or diseases. The question is asking for the most likely cause of her symptoms given the options. PLANNING: The primary assumption here is that the woman's condition is related to the options provided. Each option presents unique symptoms and characteristics. Atrial septal defect could result in pulmonary hypertension, but the patient's irregular heart rhythm may suggest a more complex condition. Chronic mitral regurgitation and rheumatic heart disease could present similar findings, but we don't have any obvious history of a rheumatic fever. Coarctation of the aorta and congenital aortic stenosis are both structural heart defects, however, they usually present differently. Mitral stenosis complicated by atrial fibrillation could also precipitate a similar clinical picture, especially if cardiac decompensation is present. CLINICAL REASONING: Given the findings of heart palpitations, irregular rhythm, and diastolic murmur, it may point to a pathologic state of the mitral valve. Furthermore, the indistinct point of maximal impulse suggests an irregularly enlarged left atrium, potentially due to mitral stenosis (narrowing of the mitral valve). Also, the snapping sound usually suggests an abrupt halt to the mitral valve leaflet motion in diastole, secondary to the increased pressure gradient across the stenotic valve. This, combined with the patient's symptoms, is consistent with mitral stenosis. The fact that it's "complicated by atrial fibrillation" further indicates the patient's irregular heart rhythm and contributes to her symptoms. CONCLUDING: Based on the clinical findings presented, which include progressive dyspnea, palpitations, and irregular rhythm, along with examination findings suggestive of mitral stenosis and atrial fibrillation, conclude that "Mitral stenosis complicated by atrial fibrillation" would be the most probable cause of this patient's condition. RESPONSE: (E) Mitral stenosis complicated by atrial fibrillation is the most likely cause of the findings in this patient.

**AI_C:** RESTATING THE QUESTION: This question asks for the most likely cardiovascular diagnosis in a 31-year-old pregnant woman showing clinical signs including dyspnea, wheezing, irregularly irregular rhythm, hemoptysis, and a low-pitched diastolic murmur. In the provided options, we have various cardiovascular conditions such as atrial septal defect with pulmonary hypertension, chronic mitral regurgitation due to rheumatic heart disease, coarctation of aorta, congenital aortic stenosis and mitral stenosis complicated by atrial fibrillation. PLANNING: To find the most likely cause, I would need to explore the symptoms, signs, and medical history of the patient. I will go with the assumption that all her symptoms are related to a single diagnosis rather than multiple unrelated conditions. CLINICAL REASONING: Starting with the irregularly irregular rhythm, this strongly suggests atrial fibrillation. The low-pitched diastolic rumbling murmur audible at the apex, preceded by a distinct snapping sound, is characteristic of mitral stenosis. Her pregnancy would increase her cardiac output and exacerbate symptoms of any pre-existing mitral stenosis, as the condition narrows the mitral valve and restricts blood flow from the left atrium to the left ventricle causing back up of fluid into lungs seen as dyspnea, wheezing, decreased breath sounds, and blood-streaked sputum. Additionally, atrial fibrillation is common in mitral stenosis due to increased left atrial pressure leading to atrial enlargement. Whereas, atrial septal defect would typically present earlier in life and be associated with a fixed splitting of S2 heart sound, pulmonary hypertension would cause a loud P2, mitral regurgitation typically causes systolic murmur. Hence, they are unlikely in this case. Congenital aortic stenosis and coarctation of the aorta tend to present earlier in life and rather show features of left ventricular hypertrophy and diminished femoral pulses, which was not detected. CONCLUDING: By putting all the pieces of information together, it appears that the patient's rapid heart rate, shortness of breath, diastolic heart murmur and the irregularly irregular rhythm are symptoms of a cardiovascular condition likely caused by mitral stenosis complicated by atrial fibrillation. RESPONSE: (E) Mitral stenosis complicated by atrial fibrillation is the most likely cause of the findings in this patient.

**AI_B:** RESTATING THE QUESTION: The clinical scenario describes a 31-year-old pregnant woman at 32 weeks' gestation who is experiencing progressive respiratory distress features as dyspnea, wheezing and a cough with blood-streaked sputum. She additionally reports a racing heart. Her physical examination findings include an irregularly irregular heart rhythm, a loud S1 heart sound, a diastolic murmur detected at the apex and diminished breath sounds at the bases with bilateral crackles. The question asks which condition is most likely causing her symptoms and examination findings. PLANNING: The patient's symptoms and physical examination findings suggest a condition causing increased strain on the heart and possible left-sided heart failure. The choices provided are varied heart conditions, including congenital and acquired. The assumption might be that the most likely cause of her symptoms would be a condition that could specifically cause these findings. CLINICAL REASONING: In analyzing these options, conditions that would directly result in the described findings should be considered. Atrial septal defect with the development of pulmonary hypertension could cause an increase in pulmonary pressure and resultant respiratory symptoms, but it wouldn't typically present as an irregularly irregular rhythm or a diastolic murmur. Chronic mitral regurgitation secondary to rheumatic heart disease could result in volume overload and symptoms of heart failure, but again the irregularly irregular rhythm is not typical. Coarctation of the aorta could result in left ventricular strain and respiratory symptoms, but it is less likely in an adult, and usually presents with hypertension, not the irregular rhythm. Congenital aortic stenosis would not explain her symptoms either. However, mitral stenosis complicated by atrial fibrillation might explain all of the patient's symptoms. Mitral stenosis typically results in pulmonary congestion, which can manifest as dyspnea, wheezing and possibly blood-streaked sputum (from pulmonary edema). The atrial fibrillation can be a complication of mitral stenosis and would explain the irregularly irregular rhythm. A diastolic murmur is often associated with mitral stenosis, and can be characterized as a low-pitched, rumbling sound located at the apex. CONCLUDING: Considering the patient's symptoms, vital signs, medical history, and physical exam findings, mitral stenosis complicated by atrial fibrillation is the best fit and most likely diagnoses. RESPONSE: (E) Mitral stenosis complicated by atrial fibrillation is the most probable cause of the findings in this patient.

**AI_E:** Restating the Question: A pregnant woman, late in her third trimester, is experiencing symptoms including difficulty breathing, wheezing, a rapid heartbeat, and small amounts of blood present in her sputum. Her past medical record indicates hypothyroidism. Her physical examination reveals a high pulse, tachypnea, normal blood pressure, decreased oxygen saturation even on 100% supplemental oxygen and irregular rhythm, along with low breath sounds bilaterally, a distinct loud S1, and a diastolic murmur. The main query is about the most plausible explanation for these findings. Planning: Five cardiological conditions are proposed as possibilities: atrial septal defect with resulting pulmonary hypertension, chronic mitral regurgitation due to rheumatic heart disease, coarctation of the aorta, congenital aortic stenosis, and mitral stenosis complicated by atrial fibrillation. Assumption of each of these as the potential causative factor should be questioned in light of the given clinical picture – the patient’s symptoms, examination findings, and obstetric status. Examination of the clinical reasoning will allow us to evaluate the clinical priority of each assumption. Clinical Reasoning: Atrial septal defect often results in dyspnea and tachycardia, common symptoms in this patient. Yet such patients usually have a fixed-split S2 on auscultation, a finding which isn't indicated here. Chronic mitral regurgitation typically presents with a holosystolic murmur and not a diastolic one, which rules out this possibility. Coarctation of the aorta and congenital aortic stenosis are typically identified earlier in life, given their nature as congenital conditions, and might not present with the particular findings of this gravid woman. Mitral stenosis complicated by atrial fibrillation has the potential to manifest as dyspnea, palpitations (‘racing’ heart), and hemoptysis (blood-streaked sputum). In addition, examination findings of a loud S1, diastolic murmur heard best at the apex, and an indistinct point of maximal impulse (indicating left atrial enlargement) are all consistent with this diagnosis. Atrial fibrillation can lead to an irregularly irregular rhythm, and pregnancy can further compound the symptoms of mitral stenosis. Concluding: After a detailed examination, we can determine that the clinical picture indicates that the woman is suffering from mitral stenosis, a condition complicated by atrial fibrillation. Response: Therefore, the most likely cause of the findings in this patient is Mitral stenosis complicated by atrial fibrillation (E).

**AI_A:** RESTATING THE QUESTION: This 31-year-old woman, who is in her third pregnancy and has had two live births before, is currently 32 weeks pregnant. She has been hospitalized due to worsening shortness of breath and wheezing for the past week. She also reports a rapid heart rate and coughing up sputum streaked with blood. She has a known history of hypothyroidism, which is managed with levothyroxine. She's on IV line, and her vital signs indicate slight fever, tachycardia, tachypnea, normalish blood pressure, and slightly low oxygen saturation, even with supplemental oxygen. Lung auscultation findings and cardiac examination details suggest an abnormality in lung function and non-regular rhythm of the heart. Additionally, a noteworthy heart murmur is also detected. The fetus's heart rate is normal. The question is asking for the most likely cause of this patient's symptoms and examination findings amongst the provided choices. PLANNING: The patient presents with a combination of respiratory and cardiac symptoms, which could indicate a cardio-respiratory issue. The options given are a range of cardiac defects and diseases that could present with a variety of symptoms, including dyspnea, wheezing, tachycardia, and a distinct cardiac murmur. Each of these needs to be evaluated in the context of the patient's specific presentation and history. CLINICAL REASONING: When considering each of the possible diagnoses, we'll have to look at the individual symptoms and how they fit in with each known disease presentation. For instance, an atrial septal defect (ASD) with pulmonary hypertension is not very likely in this case as this usually presents much earlier in life, not typically first diagnosed in a 31-year-old. Chronic mitral regurgitation secondary to rheumatic heart disease is a possibility in theory, but our patient's heart manifestation doesn't exactly align with the classic presentation of rheumatic heart disease. Coarctation of the aorta commonly presents in younger age and tends to show evidence of diminished femoral pulses. Congenital aortic stenosis is generally diagnosed early in life. The final option, mitral stenosis complicated by atrial fibrillation, matches the patient's irregularly irregular heart rhythm, their pulmonary symptoms, the pronounced S1 heart sound, the snapping sound, and the diastolic murmur. Mitral stenosis could also be exacerbated by the increased blood volume during pregnancy. CONCLUDING: Assessing the patient's symptoms alongside the hallmarks of each of the potential diagnoses, it seems probable that the most likely cause of this patient's presentation is mitral stenosis complicated by atrial fibrillation. RESPONSE: Therefore, the most accurate answer, based on the available options and the clinical data provided, would be (E) Mitral stenosis complicated by atrial fibrillation.

**Facilitator** The consensus statement synthesizes the responses of AI_A, AI_B, AI_C, AI_D, and AI_E as follows: The question presents a 31-year-old pregnant woman at 32 weeks' gestation, who is experiencing progressive respiratory distress, including dyspnea, wheezing, and coughing up blood-streaked sputum. She also reports a rapid heart rate. Her physical examination findings include an irregularly irregular heart rhythm, a loud S1 heart sound, a diastolic murmur detected at the apex, and diminished breath sounds at the bases with bilateral crackles. The question asks which condition is most likely causing her symptoms and examination findings. The patient's symptoms and physical examination findings suggest a condition causing increased strain on the heart and possible left-sided heart failure. The choices provided are varied heart conditions, including congenital and acquired. The assumption might be that the most likely cause of her symptoms would be a condition that could specifically cause these findings. In analyzing these options, conditions that would directly result in the described findings should be considered. Atrial septal defect with the development of pulmonary hypertension could cause an increase in pulmonary pressure and resultant respiratory symptoms, but it wouldn't typically present as an irregularly irregular rhythm or a diastolic murmur. Chronic mitral regurgitation secondary to rheumatic heart disease could result in volume overload and symptoms of heart failure, but again the irregularly irregular rhythm is not typical. Coarctation of the aorta could result in left ventricular strain and respiratory symptoms, but it is less likely in an adult, and usually presents with hypertension, not the irregular rhythm. Congenital aortic stenosis would not explain her symptoms either. However, mitral stenosis complicated by atrial fibrillation might explain all of the patient's symptoms. Mitral stenosis typically results in pulmonary congestion, which can manifest as dyspnea, wheezing and possibly blood-streaked sputum (from pulmonary edema). The atrial fibrillation can be a complication of mitral stenosis and would explain the irregularly irregular rhythm. A diastolic murmur is often associated with mitral stenosis, and can be characterized as a low-pitched, rumbling sound located at the apex. Considering the patient's symptoms, vital signs, medical history, and physical exam findings, mitral stenosis complicated by atrial fibrillation is the best fit and most likely diagnoses. Therefore, the most likely cause of the findings in this patient is Mitral stenosis complicated by atrial fibrillation (E).
